# Drug Repurposing Using Modularity Clustering in Drug-Drug Similarity Networks Based on Drug–Gene Interactions

**DOI:** 10.3390/pharmaceutics13122117

**Published:** 2021-12-08

**Authors:** Vlad Groza, Mihai Udrescu, Alexandru Bozdog, Lucreţia Udrescu

**Affiliations:** 1 Department of Computer and Information Technology, University Politehnica of Timişoara, 300223 Timişoara, Romania; vlad.groza@student.upt.ro (V.G.); alexandru.bozdog@cs.upt.ro (A.B.); 2 Department I—Drug Analysis, “Victor Babeş” University of Medicine and Pharmacy Timişoara, 300041 Timişoara, Romania; udrescu.lucretia@umft.ro

**Keywords:** bioinformatics, drug repurposing, complex network analysis, modularity clustering, ATC code

## Abstract

Drug repurposing is a valuable alternative to traditional drug design based on the assumption that medicines have multiple functions. Computer-based techniques use ever-growing drug databases to uncover new drug repurposing hints, which require further validation with in vitro and in vivo experiments. Indeed, such a scientific undertaking can be particularly effective in the case of rare diseases (resources for developing new drugs are scarce) and new diseases such as COVID-19 (designing new drugs require too much time). This paper introduces a new, completely automated computational drug repurposing pipeline based on drug–gene interaction data. We obtained drug–gene interaction data from an earlier version of DrugBank, built a drug–gene interaction network, and projected it as a drug–drug similarity network (DDSN). We then clustered DDSN by optimizing modularity resolution, used the ATC codes distribution within each cluster to identify potential drug repurposing candidates, and verified repurposing hints with the latest DrugBank ATC codes. Finally, using the best modularity resolution found with our method, we applied our pipeline to the latest DrugBank drug–gene interaction data to generate a comprehensive drug repurposing hint list.

## 1. Introduction

The growth in the number of newly approved pharmaceutical substances has stagnated despite the ever-growing resources that the industry allocates [1,2,3,4]. Designing, developing, and testing new medicines is an expensive, long, and cumbersome process [5], which becomes explicitly bothersome for new rare diseases—because funds are limited—and new pathogen epidemics—stopping the disease spread requires a rapid therapeutic solution [6,7]. One convenient alternative to the pharmaceutic industry’s productivity challenges is drug repurposing, underpinned by the R&D in the pharmaceutical industry, as well as the observations and long-time experience indicating the favorable polypharmacological profile of drugs (in other words, most pharmaceutical substances tend to have multiple functions) [8,9,10]. The trend that calls for drug repurposing techniques is in sync with the recent expansion of Big Data and machine learning in genetics, biology, and medicine; therefore, we witnessed the development of a wide array of computer-based methodologies to uncover new drug repurposing [11,12,13].

A significant area in computational repurposing (or repositioning) relies on the complex network representations of various drug interaction/relationship types, e.g., drug–drug [14], drug–target [15,16,17], drug–side effect [18], drug–gene. The networks consist of nodes/edges—representing drugs, targets, genes, or side effects—and links/edges—representing interactions or other types of relationships [19]. The network of specific drug interactions allows for the characterization of a complex biological system under therapy; therefore, researchers can use computational techniques and network science principles to explore the interplay between microscale interactions and macroscale behavior [14]. An important area in network science is community/cluster detection and analysis [20,21]. The assumption is that nodes from a distinct cluster have similar topological properties and, thus, share a common feature; this results in drug repurposing opportunities [6]. (If most drugs in a cluster have a particular therapeutic function, then it is reasonable to assume that the function also exists at least in some of the other drugs in the cluster). Many network-based computational drug repurposing methods use topological network features, such as centralities (topological indicators/measures of a node’s importance in the network) and modularity, to identify potential repositioning [22,23].

All computational drug repositioning methods produce lists of hints or predictions that require testing or confirmation in silico (e.g., molecular docking) [24], in vitro, and in vivo [25]. One can also indirectly prove the effectiveness of the computational technique by applying it on an earlier database version and testing the predictions on the latest data [14,22]. The existing computational pipelines predicted several important drug repurposings. Moreover, the crisis generated by the COVID-19 pandemic called for drug repurposing solutions to counter SARS-CoV-2 infections.

In our prior study, we also approached the problem of drug repositioning by building a drug–drug interaction network [14] and a drug–drug similarity network based on drug–target interactions [22]; we used the corresponding drug–drug and drug–target interaction data from DrugBank 4.1 and 4.2, respectively. In [14], we used community detection with energy-based layouts and fixed modularity; in [22], we also used energy-based layouts and fixed modularity, as well as ranking nodes by network centralities; in both previous approaches, we labeled the clusters and confirmed predictions with expert analysis.

In this paper, we also use a method based on network community detection and analysis. To this end, we build a drug–drug similarity network, because similarity networks are better suited for community detection: Nodes in the same community are more likely to be similar. Indeed, many other computational drug repurposing methods operate on similarity networks [26,27], with similarity defined on various criteria—from drug–target interactions [22] to adverse effects [18]. We find inspiration in the diseasome project [28,29] based on processing a disease–gene bipartite network (i.e., with two types of nodes, namely, genes and diseases); the processing of the disease–gene network projects it as either a gene–gene similarity or a disease–disease similarity network. In the gene–gene network, a link between two genes exists if there is at least one common disease with which they interact; in the disease–disease network, a link between two diseases exists if at least one gene is responsible for both diseases.

**Figure 1:**
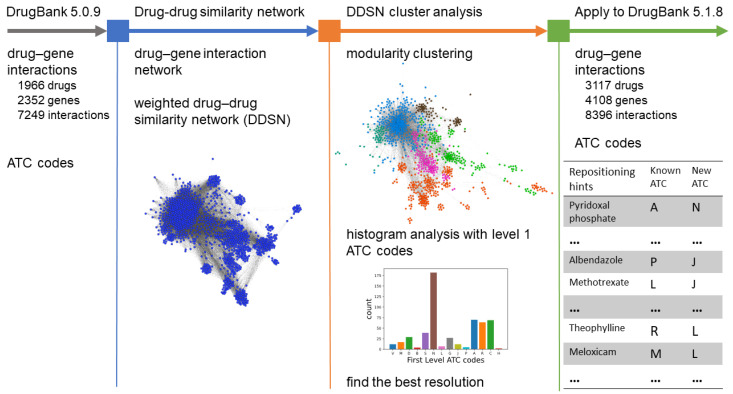
The overview of our proposed computational drug repurposing pipeline. In the first step, we use drug–gene interaction information from DrugBank 5.0.9 to build the (bipartite) drug–gene interaction network, which we then projected as a drug–drug similarity network (DDSN). In the second step, we used modularity class network clustering to identify drug communities with shared properties, analyzed the DrugBank 5.0.9 first-level ATC code histograms in each community to predict new drug properties, and checked these predictions against the latest DrugBank 5.1.8 level 1 ATC codes. The procedure in the second step allows maximizing the number of confirmed repositionings by adjusting modularity resolution. The third step uses our method with the optimized resolution value determined in the second step to generate a repurposing hints list according to DrugBank 5.1.8.

Our method builds a drug–gene interaction network with drug–gene interaction data from the earlier DrugBank 5.0.9 version, then projects it as a drug–drug similarity network; this is the first drug repurposing method derived from a gene-based drug–drug similarity network to the best of our knowledge. Our drug–drug similarity network is weighted—the weight of the link between two nodes/drugs represents the number of genes with which the two drugs interact in the same manner. We then use modularity-based network clustering to identify drug communities/clusters. We adopt the same assumption as in the case of the diseasome analysis in [30] that nodes inside the same community most probably share a common function or property. In this manner, if a drug inside one community does not have the ATC code level 1 of the majority, then we hypothesize that the drug can be repurposed accordingly. Nonetheless, we improve the efficiency of the approach by providing an automated procedure for tuning modularity resolution [31] by comparing the ATC code level 1 predicted with our method applied to DrugBank 5.0.9 [32] with the level 1 ATC codes of the drug in the latest DrugBank version 5.1.8 [33]. Finally, we apply our pipeline—with the optimized modularity resolution—to the latest DrugBank data to generate a new list of repurposing hints, which we support by existing literature findings. Refer to the overview of our proposed methodology in Figure 1. We only considered drugs listed as *approved* in DrugBank.

Three arguments support the novelty of the research presented in this paper. First, this manuscript is—to the best of our knowledge—the first to build and process a DDSN based on drug–gene interaction data. Second, we present a novel method (based on level 1 ATC codes) that labels clusters and generates repositioning hints automatically. Third, we tuned modularity resolution algorithmically and automatically confirmed repositioning hints by comparing two chronologically distinct DrugBank versions.

From a pharmacological perspective, our overarching contribution is to develop, for the first time, and promote the drug–gene interaction networks as a valuable analytical, screening, and visualization tool in drug repositioning. Our method can complement existing computational repositioning pipelines; therefore, it can be integrated into more sophisticated ensemble methods.

## 2. Materials and Methods

In this section, we present the conceptual description of our algorithmic drug repositioning method from Figure 1. The thorough technical implementation and description are provided on our GitHub page https://github.com/GrozaVlad/Drug-repurposing-using-DDSNs-and-modularity-clustering (last commit on 21 October 2021). We used *Nodejs* with packets *xml-js* (for parsing the DrugBank xml files) and *pg* (for interacting with the *PostgreSQL* database), and *Docker* and *Docker-compose* for containerized databases [34]. For building and clustering DDSN, we used the *Python* packages *Psycopg2*, *Pandas* [35], *NetworkX* [36], and *Cdlib* [37]; for visualizing the networks, we used *Gephi* [38]. The hardware platform for running this project was a MacBook Pro, Intel Core i9—2400 MHz with 16 GB RAM, GPU Radeon Pro 560× 4 GB.

### 2.1. Databases

In order to facilitate an automated procedure of validating our drug repurposing pipeline, we used the earlier DrugBank version 5.0.9 to generate repurposing predictions in one of the anatomical or pharmacological groups described by the first-level ATC codes, then we validated the predictions with the ATC codes with the latest DrugBank version 5.1.8 (last accessed on 30 September 2021).

In DrugBank version 5.0.9, there are 1966 drugs, 2352 genes, and 7249 drug–gene interactions; the interaction types are part of the set $I_{e}=$ {inhibitor, agonist, antagonist, other/unknown, ligand, partial agonist, inducer, other, suppressor, binder, antibody, modulator, allosteric modulator, potentiator, neutralizer, stimulator, activator, component of, substrate, inactivator, blocker, antisense oligonucleotide}. In the latest DrugBank version 5.1.8, there are 3117 drugs, 4108 genes, and 8396 drug–gene interactions with interaction types part of the set $I_{l}$ = {inhibitor, agonist, antagonist, other/unknown, antibody, substrate, ligand, partial agonist, inducer, other, suppressor, binder, potentiator, modulator, activator, cofactor, degradation, positive allosteric modulator, incorporation into and destabilization, allosteric modulator, neutralizer, stimulator, binding, inactivator, inverse agonist, blocker, chaperone, inhibition of synthesis, antisense oligonucleotide, gene replacement, regulator}. Refer to Section 4.1 for explanations.

We chose DrugBank [33] because it is a comprehensive, versioned, and scientifically curated (i.e., robust) database with consistent support for in silico drug design and repositioning space exploration [32].

### 2.2. Building the Drug–Drug Similarity Network

**Figure 2:**
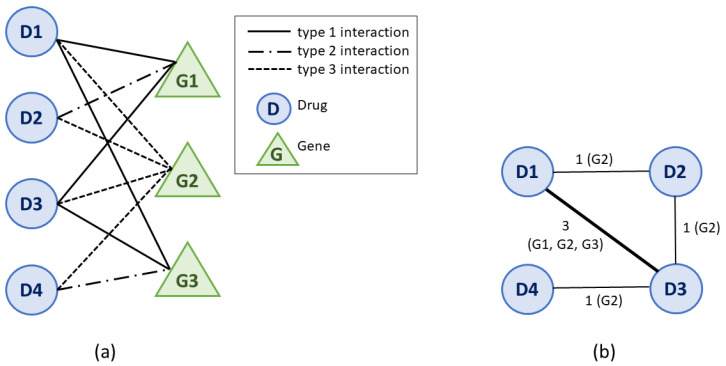
An illustrative example of projecting the bipartite drug–gene interaction graph G (**a**) into a weighted drug–drug similarity network W (**b**). In our example, G has 4 drugs ($D_{1}$, $D_{2}$, $D_{3}$, and $D_{4}$), 3 genes ($G_{1}$, $G_{2}$, and $G_{3}$), and 3 types of drug–gene interactions. In the drug–drug similarity network from panel (**b**), nodes are drugs, and links between two drugs represent the number of genes with which the drugs interact in the same manner. For instance, as shown, the link $w_{1,3}$ between nodes/drugs $D_{1}$ and $D_{3}$ has a weight of 3 because $D_{1}$ and $D_{3}$ have the same type of interaction with genes $G_{1}$, $G_{2}$, and $G_{3}$.

The bipartite drug–gene interaction network is a graph $G=\left(V,E\right)$, where *V* is the set of vertices or nodes, and *E* is the set of edges. The network G is bipartite because $V=V_{D}\cup V_{G}$, where $V_{D}$ is the set of drugs and $V_{G}$ is the set of genes. The edges $e_{ij}\in E$ represent interactions between a drug $D_{i}\in V_{D}$ and a gene $G_{j}\in V_{G}$ (the interaction is of the type $T_{k}\in I$, with *I* defined in Section 2.1). An example of such a drug–gene bipartite graph is presented in Figure 2a, with 4 drugs, 3 genes, and 3 types of drug–gene interactions.

From the drug–gene bipartite network G, we generated the weighted drug–drug similarity network $W=\left(V_{D},W\right)$ using network projection [39]. In the DDSN, the nodes represent drugs, and a link between two nodes exists if there is at least one gene with which the two drugs interact in the same manner (i.e., the interactions are of the same type $T_{k}\in I$). In Figure 2b, we present the DDSN projection of the drug–gene example network in Figure 2. The network is weighted because two drugs $D_{i}$ and $D_{j}$ can have the same type of interactions with *m* genes; therefore, the weight of edge $w_{ij}\in W$ is *m*.

### 2.3. Network Clustering Analysis

The clustering of network $G=\left(V,E\right)$ is the process of classifying all nodes $v_{i}\in V$ in one of the *n* (disjoint) subsets $C_{j}$, with $V={⋃}_{j=1}^{n}C_{j}$, according to their topological properties. In this paper, we use modularity-based clustering because of its proven effectiveness in drug network analysis [14,22,23]. As defined in [40], the modularity of a clustering C in a weighted network such as our DDSN—represented as W—is defined as follows.

(1) $$M=\frac{1}{2a}\sum_{ij}\left(w_{ij}-\frac{k_{i}k_{j}}{2a}\right)p\left(C_{i},C_{j}\right).$$

In Equation (1), $a=\frac{1}{2}\sum_{ij}w_{ij}$; *i* and *j* are the indexes of nodes $v_{i},v_{j}\in V_{D}$; $k_{i}$ and $k_{j}$ are the node degrees (i.e., the sums of weights of incident edges) for nodes $v_{i},v_{j}\in V_{D}$; $w_{ij}$ is the adjacency matrix of nodes in W; $C_{i}$ and $C_{j}$ are the communities that include nodes $v_{i},v_{j}\in V_{D}$, respectively; and *p* is a function $p\left(x,y\right)$ that returns 1 if x=y and 0 otherwise. (In our DDSN, nodes $v_{i}$ and $v_{j}$ are drugs $D_{i}$ and $D_{j}$, respectively).

The modularity of clustering C is a value $M_{C}\in \left[-1,1\right]$, representing the edge density within the clusters with respect to the edge density between clusters. The clustering algorithms are based on modularity search for the best partitioning C of the node-set such that the value of *M* is maximized. The problem is that an exhaustive search for the best modularity entails large computational burden. Consequently, in practice, heuristic algorithms approximate optimal modularity clustering. However, if the network is very large, such approximations cannot identify small-size clusters—even if the density of internal edges is high and the density of edges between these small clusters and the rest of the network is low.

In this paper, we use the modularity-based clustering algorithm from [41], which controls the resolution of the clustering using a recursive procedure that starts with each node being a cluster and then moving nodes $v_{i}$ (i.e., $D_{i}$ in our DDSN) to a different cluster $C_{j}$ if this generates a positive modularity gain expressed as follows.

(2) $$\Delta M=\left[\frac{K_{C_{j}}^{*}+K_{i}^{C_{j}}}{2a}-{\left(\frac{K_{C_{j}}+K_{i}}{2a}\right)}^{2}\right]-\left[\frac{K_{C_{j}}^{*}}{2a}-{\left(\frac{K_{C_{j}}}{2a}\right)}^{2}-{\left(\frac{K_{i}}{2a}\right)}^{2}\right].$$

In Equation (2), $K_{C_{j}}^{*}$ is the sum of the weights of all edges within cluster $C_{j}$; $K_{C_{j}}$ is the sum of the weights of all edges incident to nodes in cluster $C_{j}$; $K_{i}$ is the sum of the weights of all edges incident to node $v_{i}$ ($D_{i}$ in DDSN); and $K_{i}^{C_{j}}$ is the sum of the weights of links from $v_{i}$ to all nodes in cluster $C_{j}$. The algorithm controls the clustering resolution using the value of λ=ΔM—a lower λ determines a higher number of clusters.

### 2.4. Tuning Resolution λ

Using Algorithm 1, we tune the modularity resolution to achieve efficiency in predicting new drug properties. To this end, we try λ values in the $\left[0.1,5\right]$ interval, with a step of 0.1, generate the modularity clustering C for each resolution value ($\mathrm{Clustering}\left(G,\lambda \right)$), and determine the dominant property $P_{i}$ in each cluster $C_{i}\in C$. The dominant property $P_{i}$ corresponds to the level 1 ATC code of the majority of drugs in cluster *i*, $D_{j}\in C_{j}$, as resulting from the level 1 ATC code histogram of $C_{i}$, and denoted $A^{1}\left(C_{i}\right)$. Then, for each drug $D_{j}$ in each cluster $C_{i}$, we checked the list of first level ATC codes for drug $D_{j}$ (denoted $A^{1}\left(D_{j}\right)$) against the drug’s cluster dominant property $P_{i}$. If $P_{i}$ is not in the list of DrugBank 5.0.9 level 1 ATC codes for $D_{j}$ (i.e., $A^{1}\left(D_{j}\right)$), but it is present in the list of DrugBank 5.1.8 level 1 ATC codes (i.e., $A_{c}^{1}\left(D_{j}\right)$), then we consider this as a confirmed repositioning of $D_{j}$ to property $P_{i}$. As such, we will add drug $D_{j}$ to the list of repositionings confirmed with DrugBank 5.1.8 level 1 ATC codes, $R^{c}$. Value $\lambda_{max}$ corresponds to $R^{c}$ with the biggest number of elements, namely $\max\left\{|R^{c}|\right\}$.
**Algorithm 1** Find the parameter λ, such that the clustering C of nodes/drugs $D_{i}$ in G with modularity resolution λ (i.e., $\mathrm{Clustering}\left(G,\lambda \right)$) produces the biggest number of repositionings confirmed with the level 1 ATC codes in DrugBank 5.1.8.**Input:** Drug-drug similarity network $G=\left(V_{D},E\right)$ based on drug-gene interaction data from DrugBank 5.0.9., ATC codes for drugs in DrugBank versions 5.0.9 and 5.1.8**Output:** The λ value that generates the highest number of confirmed repositionings.1:**for**λ in range (0.1 to 5), with 0.1 steps **do**2:    $C⇐\mathrm{Clustering}\left(G,\lambda \right)$3:    **for all** $C_{i}\in C$ **do**4:        $P_{i}⇐A^{1}\left(C_{i}\right)$5:        $R_{i}^{c}⇐\emptyset$6:        **for all** $D_{j}\in C_{i}$ **do**7:           **if** $\;\mathrm{then}P_{i}\notin A^{1}\left(D_{j}\right)\;\&\;P_{i}\in A_{c}^{1}\left(D_{j}\right)$8:               $R_{i}^{c}⇐R_{i}^{c}\cup \left\{D_{j}\right\}$9:           **end if**10:        **end for**11:    **end for**12:    $R^{c}=⇐{⋃}_{i}R_{i}^{c}$13:**end for**14:**Return** the value of $\lambda_{max}$ corresponding to $\max\left\{|{ʹR}^{c}|\right\}$

### 2.5. Generating New Repurposing Hints

We generated a list of new repositioning hints using the modularity clustering with the resolution value determined by Algorithm 1 in Section 2.4. Algorithm 2 presents the method we follow: Cluster the DDSN built with drug–gene interaction information from DrugBank 5.1.8 using the tuned resolution $\lambda_{max}$ ($C=\mathrm{Clustering}\left(G,\lambda_{max}\right)$); determine the dominant property $P_{i}$ of each cluster $C_{i}\in C$ as resulted from $C_{i}$’s level 1 ATC code histogram (denoted $A^{1}\left(C_{i}\right)$); and check for each drug $D_{j}$ in each cluster $C_{i}$ the list of first level ATC codes of $D_{j}$ (denoted $A^{1}\left(D_{j}\right)$) against its cluster’s dominant property $P_{i}$. If the cluster’s dominant property $P_{i}$ is not in $A^{1}\left(D_{j}\right)$ (the list of $D_{j}$ level 1 ATC codes), we hint that $D_{j}$ can be repositioned to $P_{i}$. Consequently, we add these repositioning cases as drug–predicted property pairs $\left(D_{j},P_{i}\right)$ to the repositioning hints list N.
**Algorithm 2** Generate the list of drug repurposing hints by clustering the DDSN G with the tuned modularity resolution.**Input:** Drug–drug similarity network $G=\left(V_{D},E\right)$ based on drug–gene interaction data from DrugBank 5.1.8, $\lambda_{max}$, and the ATC codes for drugs in DrugBank 5.1.8.**Output:** The repositioning hints N as a list of drug–predicted property pairs, $\left(D_{j},A^{1}\left(C_{i}\right)\right)$.1:$C⇐\mathrm{Clustering}\left(G,\lambda_{max}\right)$2:N⇐∅3:**for all**$C_{i}\in C$**do**4:    $P_{i}⇐A^{1}\left(C_{i}\right)$5:    **for all** $D_{j}\in C_{i}$ **do**6:        **if** $P_{i}\notin A^{1}\left(D_{j}\right)$ **then**7:           $N⇐N\cup \left\{\left(D_{j},P_{i}\right)\right\}$8:        **end if**9:    **end for**10:**end for**11:**Return** the list of drug repositionings N as drug–predicted property pairs

## 3. Results

### 3.1. DDSN Using Drug–Gene Interactions from DrugBang 5.0.9

**Figure 3:**
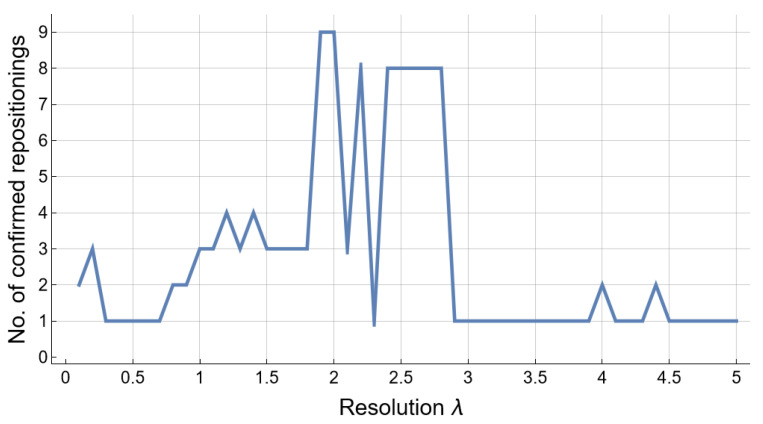
The number of confirmed repositionings $R^{c}$ for resolution λ values in the $\left[0.1,5\right]$ interval, with a step of 0.1, after running Algorithm 1 on the DDSN G built with drug–gene interaction information from DrugBank 5.0.9. The highest number of repositionings confirmed with level 1 ATC codes from DrugBank 5.1.8 (i.e., 9) corresponds to resolutions 1.9 and 2.0.

Following the algorithmic approach presented in Figure 1, according to the methods described in Section 2.2, Section 2.3, Section 2.4 and Section 2.5, we employ cluster-based network analysis on the drug–drug similarity network (DDSN) built with drug–gene interaction information from DrugBank 5.0.9 to search for the most effective modularity resolution $\lambda_{max}$—in other words, the modularity resolution that produces the highest number of drug repositionings confirmed with level 1 ATC codes from DrugBank 5.1.8. Figure 3 presents the result of running Algorithm 1 from Section 2.4; the best results correspond to resolutions 1.9 and 2.0 (the same nine confirmed repositionings in both cases). Henceforth, we will consider $\lambda_{max}=2.0$.

**Figure 4:**
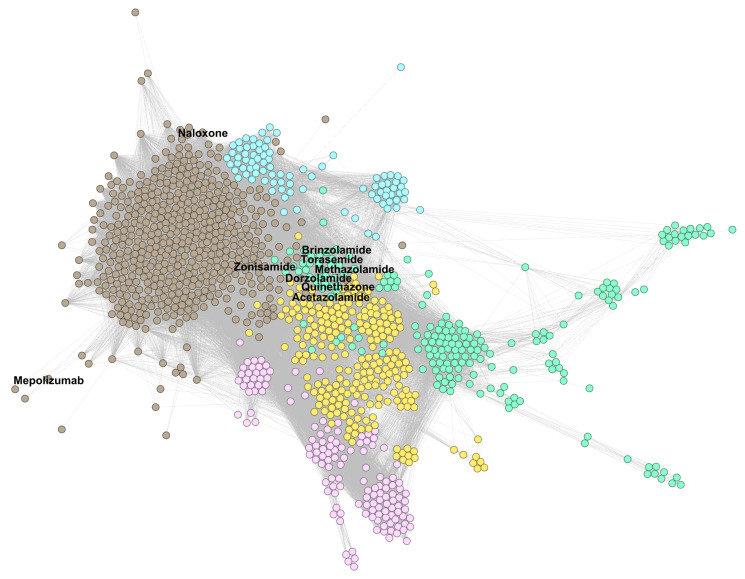
Drug–drug similarity network (DDSN) built with drug–gene interaction data from DrugBank 5.0.9, clustered using modularity classes for resolution $\lambda_{max}=2.0$. We indicate the position of drugs repositioned and confirmed (with level 1 ATC codes from DrugBank 5.1.8) them by labeling the corresponding nodes with their names. The brown nodes represent drugs in cluster $C_{0}$ (512 drugs), yellow nodes represent drugs in cluster $C_{1}$ (238 drugs), green nodes represent drugs in cluster $C_{2}$ (197 drugs), pink nodes represent drugs in cluster $C_{3}$ (143 drugs), and light blue nodes represent drugs in cluster $C_{4}$ (88 drugs).

Figure 4 presents the largest connected component of the DDSN, constructed with drug–gene interaction data from DrugBank 5.0.9 and clustered with modularity resolution $\lambda_{max}=2.0$; the text indicates the topological coordinates of repositionings confirmed with DrugBank 5.1.8 data.

In Figure 4, nodes represent drugs, and links represent similarity relationships based on drug–gene interactions, as described in Section 2.2; node colors correspond to specific clusters, as determined by the modularity class, and all links are represented with grey lines.

**Figure A1:**
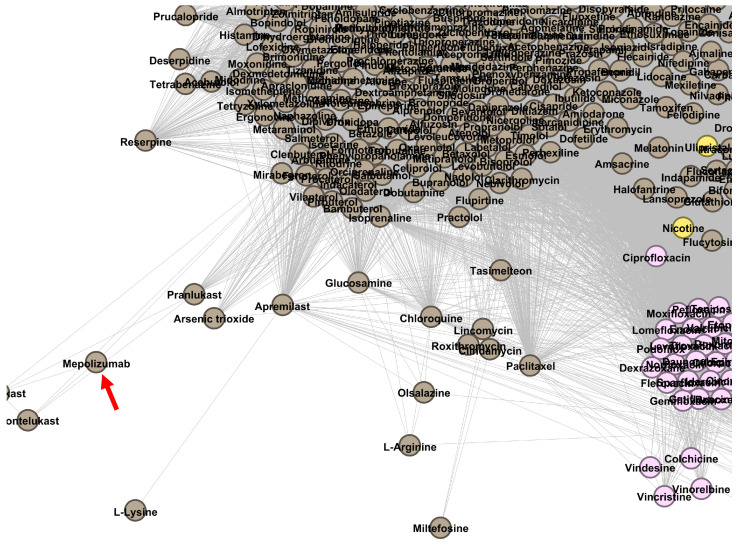
The zoomed detail of the DDSN network built with drug–gene interaction data from DrugBank 5.0.9, which shows the relative position of mepolizumab within cluster $C_{0}$ (brown nodes) with a red arrow (→). Our repositioning pipeline predicts that mepolizumab—listed as antineoplastic in DrugBank 5.0.9—also acts as a drug with level 1 ATC code R (*Respiratory system*), confirmed by the more recent DrugBank version 5.1.8.

**Figure A2:**
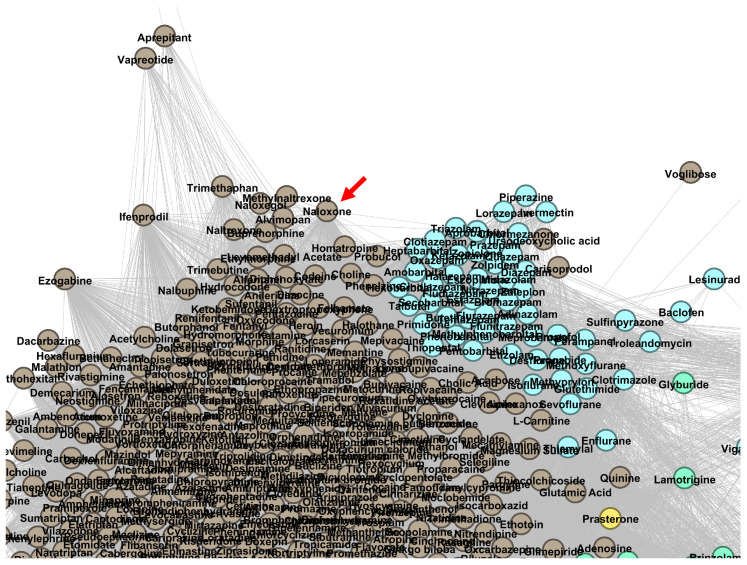
The zoomed detail of the DrugBank 5.0.9 DDSN network showing the relative position of naloxone within cluster $C_{0}$ (brown nodes) with a red arrow (→). Our repositioning pipeline predicts that naloxone—listed as opioid overdose antidote in DrugBank 5.0.9—also acts as a drug with level 1 ATC code N (*Nervous system*), confirmed by the more recent DrugBank version 5.1.8.

**Figure A3:**
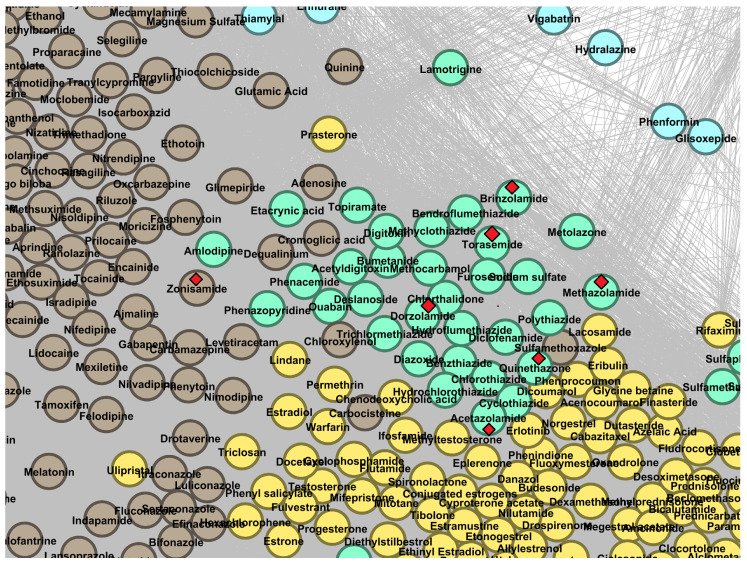
The DrugBank 5.0.9 DDSN network’s zoomed detail shows the confirmed repositionings within cluster $C_{2}$ (green nodes) with red diamonds (⋄). Our repositioning pipeline predicts that torasemide and quinetazone (both with ATC level 1 code C–*Cardiovascular system* in DrugBank 5.0.9), methazolamide, acetazolamide, dorzolamide, and brinzolamide (all with ATC level 1 code S–*Sensory organs* in DrugBank 5.0.9) are *Genito urinary system and sex hormones* drugs (first level ATC G). Zonisamide (N–*Nervous system*) is a brown node (cluster $C_{0}$) but in the close vicinity of cluster $C_{2}$; therefore, they are also predicted at level 1 ATC code G.

In Appendix A.1, Figure A1, Figure A2 and Figure A3, present zoomed details of DDSN from Figure 4 in the vicinity of nine confirmed repositionings corresponding to $\lambda_{max}=2.0$. The repositionings come from cluster $C_{0}$–brown and cluster $C_{2}$–green nodes. We indicated the drug repositionings confirmed with DrugBank 5.1.8 data with red arrows (→) in Figure A1 and Figure A2; in Figure A3, we have many confirmed repurposed drugs and a high density of nodes; hence, red diamonds (⋄) were used instead of arrows.

**Figure A4:**
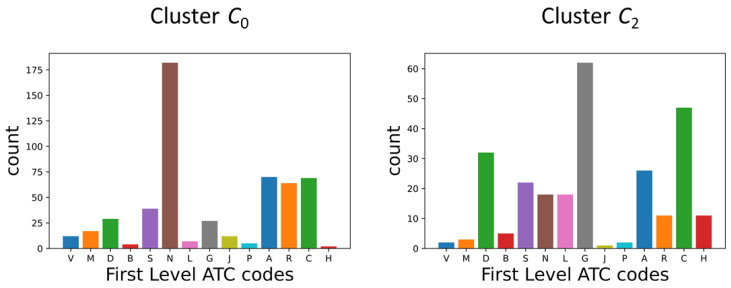
Histograms of level 1 ATC codes in the DrugBank 5.0.9 DDSN clusters holding drug repositionings confirmed by DrugBank 5.1.8: cluster $C_{0}$ (brown nodes) in the left panel and cluster $C_{2}$ (green nodes) in the right panel. The dominant property in cluster $C_{0}$ is N–*Nervous system*, with many subcluster drugs with level 1 ATC codes A, R, and C (*Alimentary tract and metabolism*, *Respiratory system*, and *Cardiovascular system*, respectively). The dominant properties in cluster $C_{2}$ are G, C, and D (*Genito urinary system and sex hormones*, *Cardiovascular system*, and *Dermatologicals*, respectively).

The zoomed details provided by Figure A1 and Figure A2 show that mepolizumab and naloxone are within cluster $C_{0}$ (brown nodes), where the dominant property is given by the level 1 ATC code N–*Nervous system*, followed by code R–*Respiratory system*. As such, our method automatically predicts that mepolizumab (listed as L–*Antineoplastic and immunomodulatory drugs* in DrugBank 5.0.9) acts as a drug with level 1 ATC code R. (In Appendix A.2, Figure A4 shows that in cluster $C_{0}$—in addition to the dominant level 1 ATC codes N—we also have many subcluster drugs with level 1 ATC codes A–*Alimentary tract and metabolism*; R–*Respiratory system*; and C–*Cardiovascular system*). Our method predicts that naloxone (an opioid overdose antidote in DrugBank 5.0.9) also acts on the nervous system (first level ATC N). The more recent DrugBank 5.1.8 confirms the predictions, listing mepolizumab with first level ATC code R and naloxone with N (see more details in Section 3.3.1).

In Appendix A.1, Figure A3, we zoom in to the region in DrugBank 5.0.9 DDSN with the confirmed repositionings in cluster $C_{2}$ (green nodes), with the dominant level 1 ATC code G–*Genitourinary system and sex hormones* (see the histogram in Appendix A.2 Figure A4). The confirmed repositionings in cluster $C_{2}$ are torasemide (ATC level 1 code C, cardiovascular system), quinetazone (C), methazolamide (S, sensory organs), acetazolamide (S), dorzolamide (S), and brinzolamide (S). Zonisamide (N, nervous system) is a brown node (cluster $C_{0}$) but in the close vicinity of cluster $C_{2}$; therefore, one can expect functional overlappings [14]. Our method automatically predicts that all these drugs have genitourinary system properties, and DrugBank 5.1.8 confirms the predictions (see the detailed description in in Section 3.3.1).

Using ATC codes as references for drug repurposing is already used in the state-of-the-art contexts, although confirmations based on ATC codes are very conservative (i.e., the World Health Organization assigns new ATCs after a long and thorough process) [25,42]. Confirming the predicted drug repositionings by performing a research literature review will reveal many more confirmations [25,43]. By this logic, our analysis of DrugBank 5.0.9 does not reveal many confirmed repurposings, yet it helps tune the modularity resolution λ.

### 3.2. DDSN Using Drug–Gene Interactions from DrugBang 5.1.8

**Figure 5:**
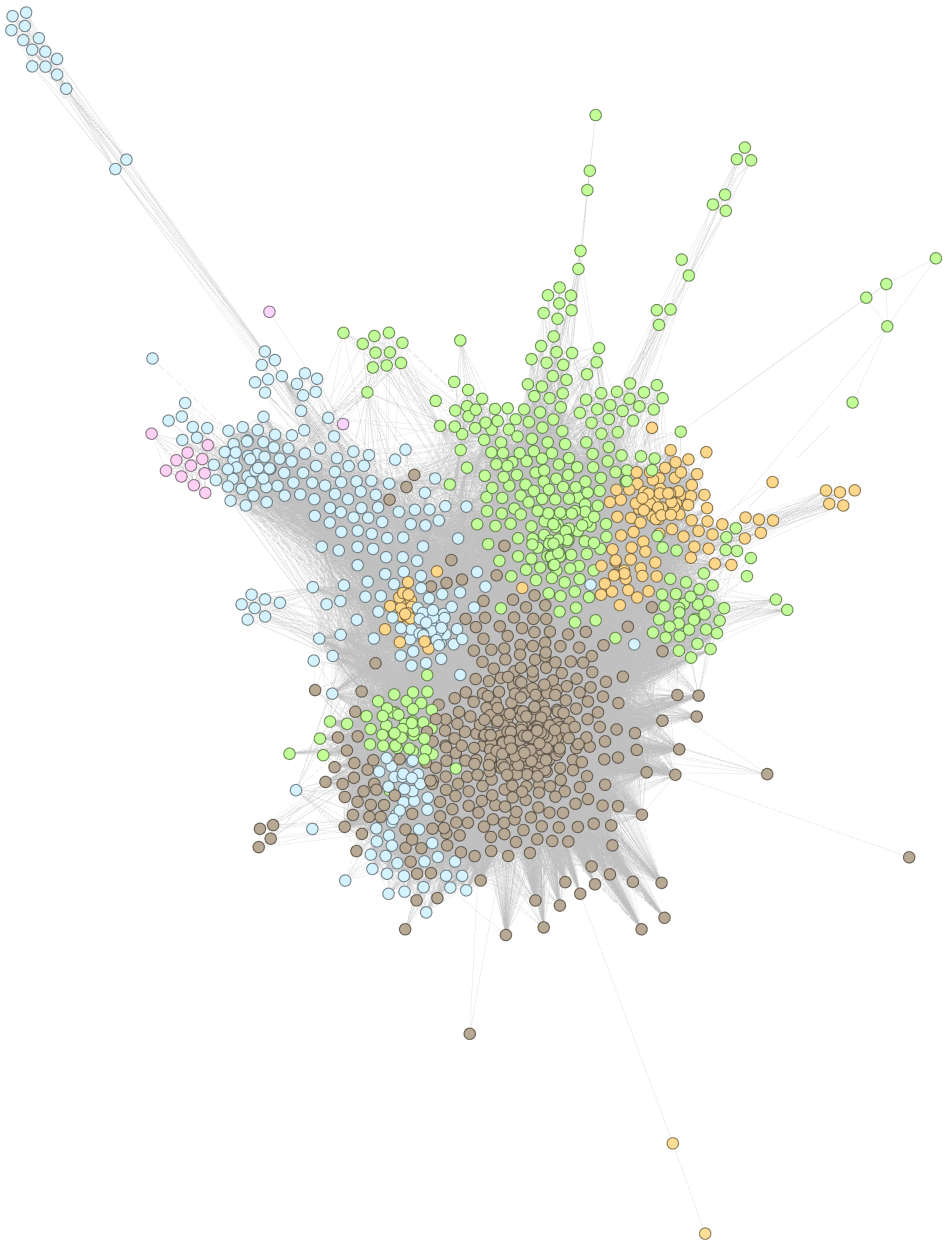
Drug–drug similarity network (DDSN) built with drug–gene interaction data from DrugBank 5.1.8, clustered using modularity classes for resolution $\lambda_{max}=2.0$. The brown nodes represent drugs in cluster $C_{0}$ (479 drugs), green nodes represent drugs in cluster $C_{1}$ (346 drugs), light blue nodes represent drugs in cluster $C_{2}$ (270 drugs), orange nodes represent drugs in cluster $C_{3}$ (129 drugs), and pink nodes represent drugs in cluster $C_{4}$ (12 nodes).

According to the algorithmic approach presented in Figure 1, we generated the DDSN based on the drug–gene interactions reported in DrugBank 5.1.8 and clustered DDSN using the modularity classes obtained for resolution $\lambda_{max}$ (by employing Algorithm 1 with the results presented in Section 3.1). We display the largest connected component of the DrugBank 5.1.8 DDSN in Figure 5, with cluster $C_{0}$ (brown nodes) having the dominant level 1 ATC code N–*Nervous system*; clusters $C_{1}$ and $C_{2}$ (green and orange nodes) J–*Anti-infectives for systemic use*; cluster $C_{3}$ (light blue nodes) L–*Antineoplastic and immunomodulating agents*; and cluster $C_{4}$ (pink nodes) A–*Alimentary tract and metabolism*.

By running Algorithm 2 on the DDSN built with DrugBank 5.1.8 data and clustered with modularity classes at resolution $\lambda_{max}$, we generated lists of drug repurposing hints for each drug cluster. In the Supplementary Materials Table S1 file *DDSN-results.xls*, tab *DB 5.1.8 resolution 2.0*, we present the first 10 drug clusters and the entire list of drug repurposing candidates generated with Algorithm 2 (759 candidates).

Generating a list of 759 drug repurposing candidates with the latest DrugBank data and experimental confirmation is beyond the focus of our paper, and we select the first 10 drugs in each cluster in terms of betweenness/degree centrality (the methodology used in [22]) and checked them with the state-of-the-art scientific literature. For checking repositioning hints, we searched for articles in PubMed. The terms we used to search the literature were the name of the drug and the words/pharmacological terms that form level 1 of the ATC code. For example, our methodology predicted for methotrexate ATC code with level 1 J–*Anti infectives for systemic use*; we searched for the confirmation of this prediction by using keywords *methotrexate anti-infective*, as well as keywords representing therapeutic groups included in class J (i.e., *methotrexate antiviral*, *methotrexate antibacterial*, or *methotrexate antimycotic*). The confirmation results of our extensive literature check are presented in Table 1, showing the drug name, cluster number, current level 1 ATC code, predicted level 1 ATC code, and confirmation references. We also added a detailed discussion of the repurposing hints from Table 1 in Section 3.2.

**Figure 6:**
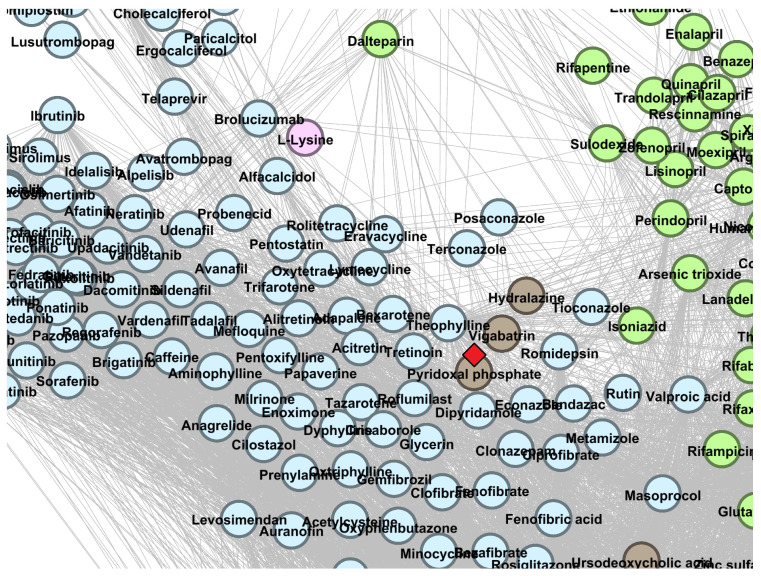
The DrugBank 5.1.8 DDSN network’s zoomed detail shows the repositioning within cluster $C_{0}$ (brown nodes) with a red diamond (⋄). Our repositioning pipeline predicts that pyridoxal phosphate (currently at ATC level 1 code A–*Alimentary tract and metabolism*) has properties described by the level 1 ATC code N—*Nervous system*.

We present the topological DDSN placement of Pyridoxal phosphate—predicted repositioning from cluster $C_{0}$—in Figure 6, where a red diamond (⋄) marks the exact position.

**Figure 7:**
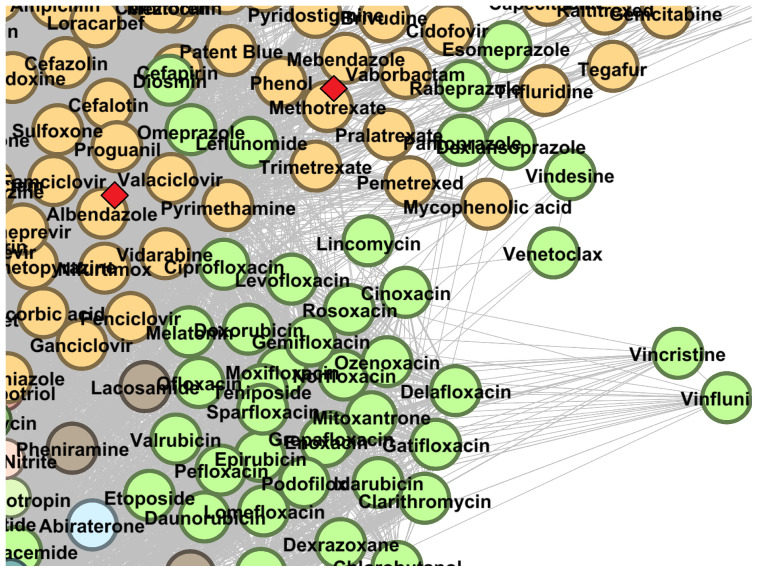
The DrugBank 5.1.8 DDSN network’s zoomed detail shows two repositionings within cluster $C_{1}$ (green nodes) with a red diamond (⋄). Our repositioning pipeline predicts that albendazole and methotrexate (currently at ATC level 1 codes P–*Antiparasitic products, insecticides, and repellents* and L–*Antineoplastic and immunomodulating agents*, respectively) have properties described by the level 1 ATC code J–*Anti infectives for systemic use*.

**Figure A5:**
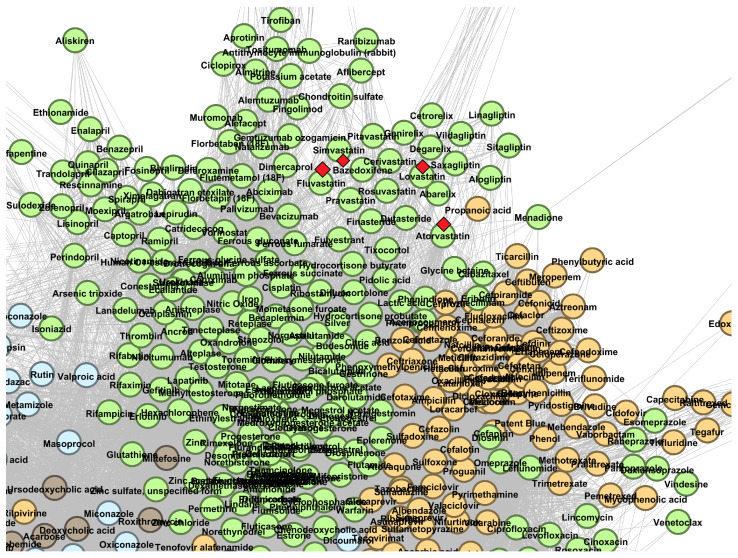
The DrugBank 5.1.8 DDSN network’s zoomed detail shows four repositionings within cluster $C_{1}$ (green nodes) with a red diamond (⋄). Our repositioning pipeline predicts that simvastatin, fluvastatin, lovastatin, and atorvastatin (currently at ATC level 1 codes C–*Cardiovascular system*) have properties described by the level 1 ATC code J–*Anti infectives for systemic use*.

In Figure 7, we illustrate the position of albendazole and methotrexate in the DDSN built with DrugBank 5.0.8 data as predicted drug repositionings from cluster $C_{1}$. Other drug repurposing candidates from cluster $C_{1}$ (presented in Table 1) are shown in Appendix B.1 and Figure A5: simvastatin, fluvastatin, lovastatin, and atorvastatin.

**Figure 8:**
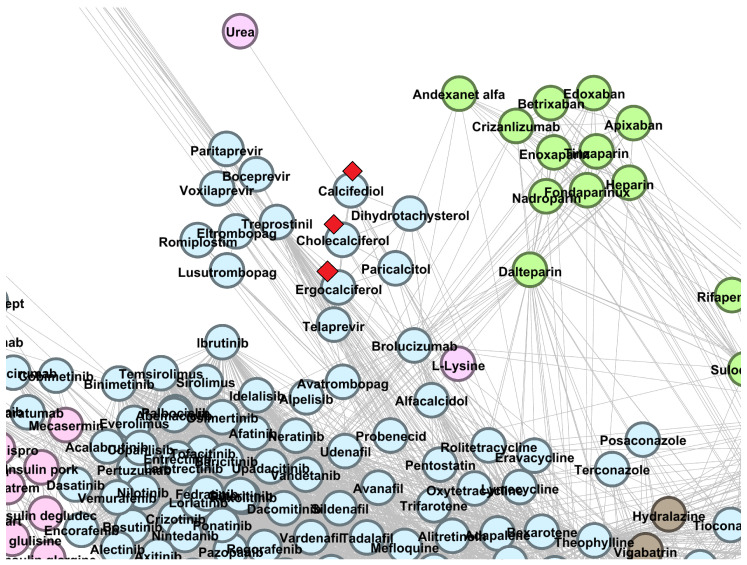
The DrugBank 5.1.8 DDSN network’s zoomed detail shows three repositionings (vitamin D derivatives) within cluster $C_{2}$ (light blue nodes) with a red diamond (⋄). Our repositioning pipeline predicts that cholecalciferol, ergocalciferol, and calcifediol (currently at ATC level 1 codes A–*Alimentary tract and metabolism* and M–*Musculo-skeletal system*) have properties described by the level 1 ATC code L–*Antineoplastic and immunomodulating agents*.

**Figure A6:**
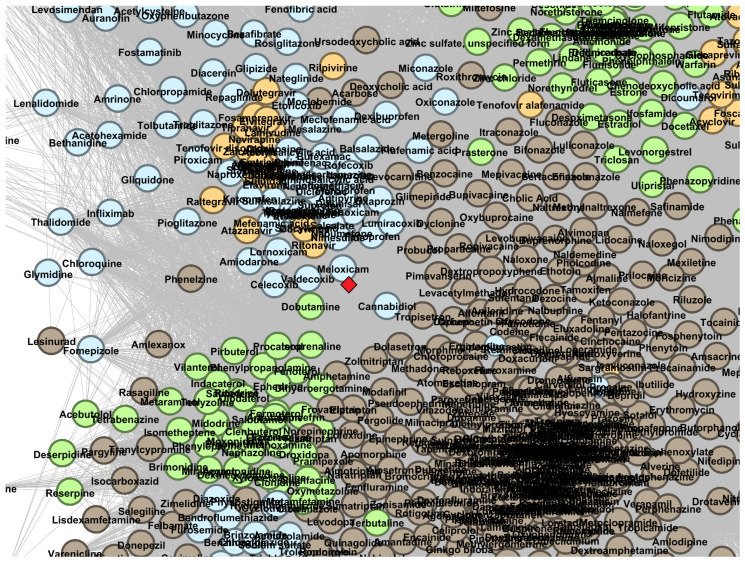
The DrugBank 5.1.8 DDSN network’s zoomed detail shows a repositionings within cluster $C_{2}$ (light blue nodes) with a red diamond (⋄). Our repositioning pipeline predicts that meloxicam (currently at ATC level 1 code M–*Musculo-skeletal system*) has properties described by the level 1 ATC code L–*Antineoplastic and immunomodulating agents*.

**Figure A7:**
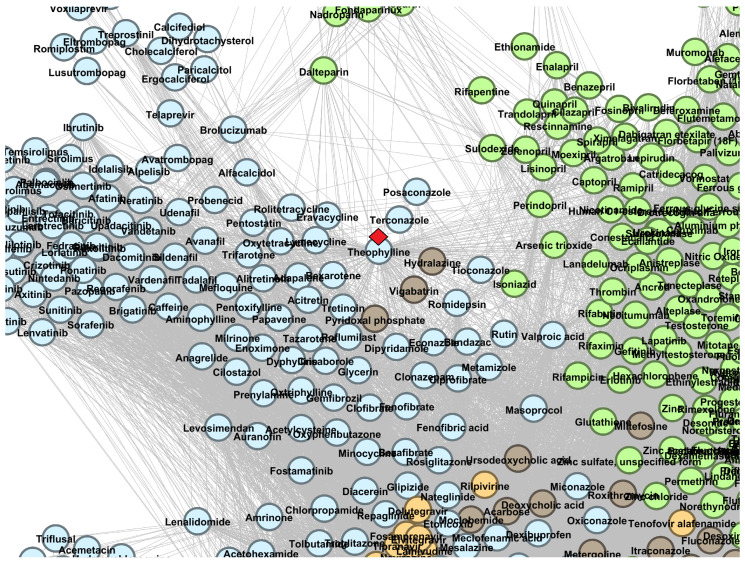
The DrugBank 5.1.8 DDSN network’s zoomed detail shows a repositioning within cluster $C_{2}$ (light blue nodes) with a red diamond (⋄). Our repositioning pipeline predicts that theophylline (currently at ATC level 1 code R–*Respiratory system*) has properties described by the level 1 ATC code L–*Antineoplastic and immunomodulating agents*.

**Figure A8:**
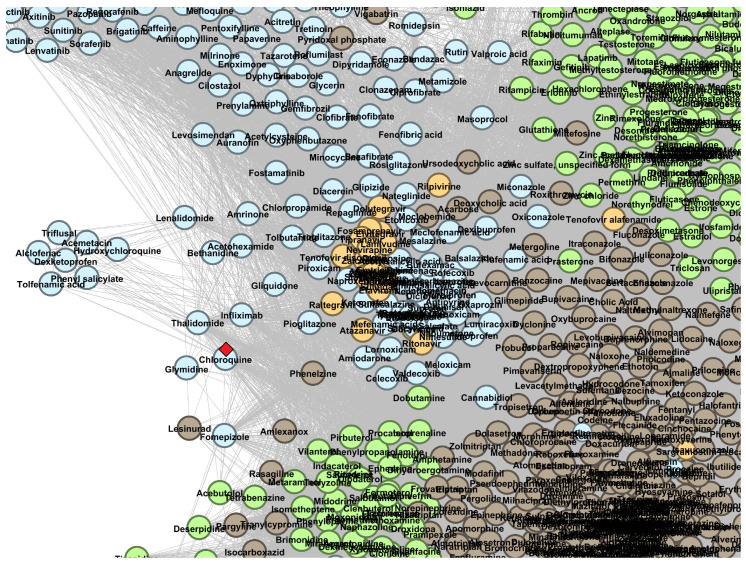
The DrugBank 5.1.8 DDSN network’s zoomed detail shows repositioning within cluster $C_{2}$ (light blue nodes) with a red diamond (⋄). Our repositioning pipeline predicts that chloroquine (currently at ATC level 1 code P–*Antiparasitic products, insecticides and repellents*) has properties described by the level 1 ATC code L–*Antineoplastic and immunomodulating agents*.

Figure 8 displays the DrugBank 5.0.8 DDSN placement of cholecalciferol, ergocalciferol, and calcifediol—drug repurposing candidates from cluster $C_{2}$. In Appendix B.1, Figure A6, Figure A7 and Figure A8, we identify the topological positions of the other drug repurposing canditates in cluster $C_{2}$ (Table 1): meloxicam, theophylline, and chloroquine.

**Figure 9:**
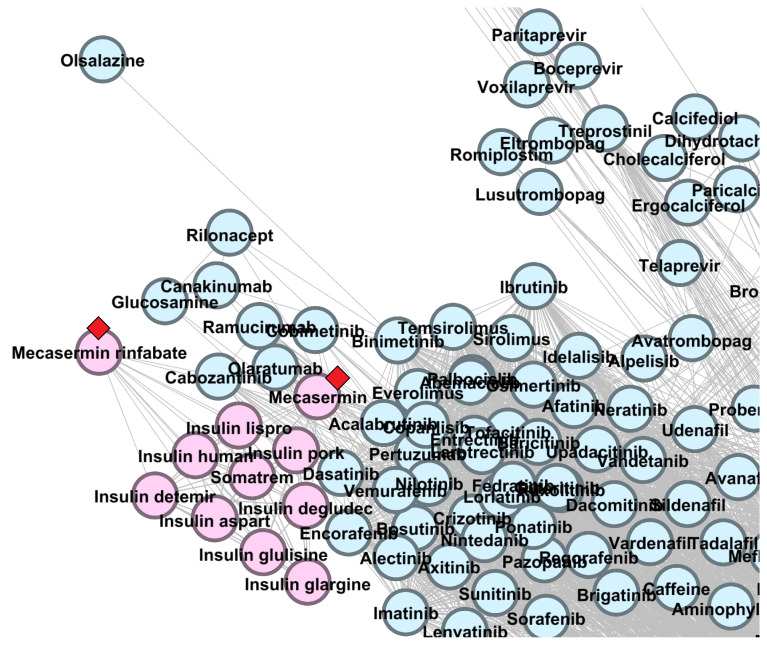
The DrugBank 5.1.8 DDSN network’s zoomed detail shows two repositionings within cluster $C_{4}$ (pink nodes) with a red diamond (⋄). Our repositioning pipeline predicts that mecasermin and mecasermin rinfabate (currently at ATC level 1 codes H–*Systemic hormonal preparations, excluding sex hormones and insulins*) have properties described by the level 1 ATC code A–*Alimentary tract and metabolism*.

**Figure 10:**
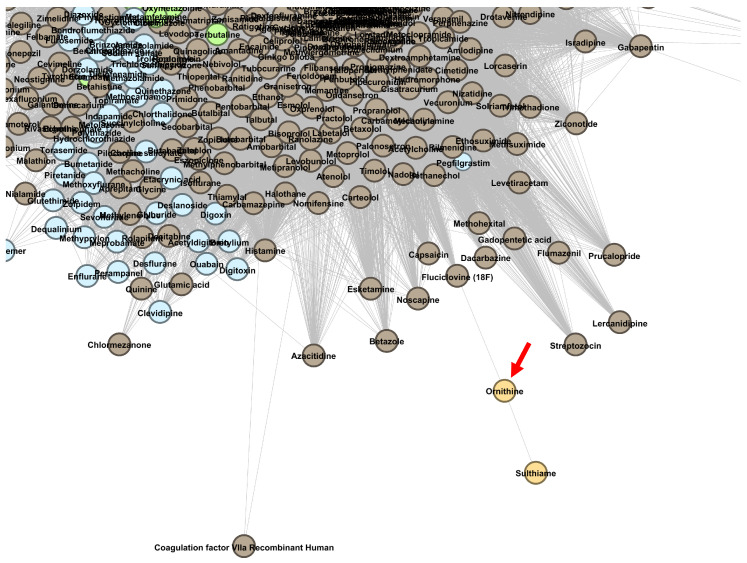
The DrugBank 5.1.8 DDSN network’s zoomed detail shows a repositioning within cluster $C_{25}$ (light orange) with a red arrow (→). Our method predicts that ornithine (currently at ATC level 1 code A–*Alimentary tract and metabolism*) has properties described by the level 1 ATC code N–*Nervous system*.

We also show the placement of drug repurposing candidates mecasermin and mecasermin rinfabate (in Figure 9, in cluster $C_{4}$, with red diamonds ⋄) and ornithine (in Figure 10, in cluster $C_{25}$, with a red arrow →).

**Figure A9:**
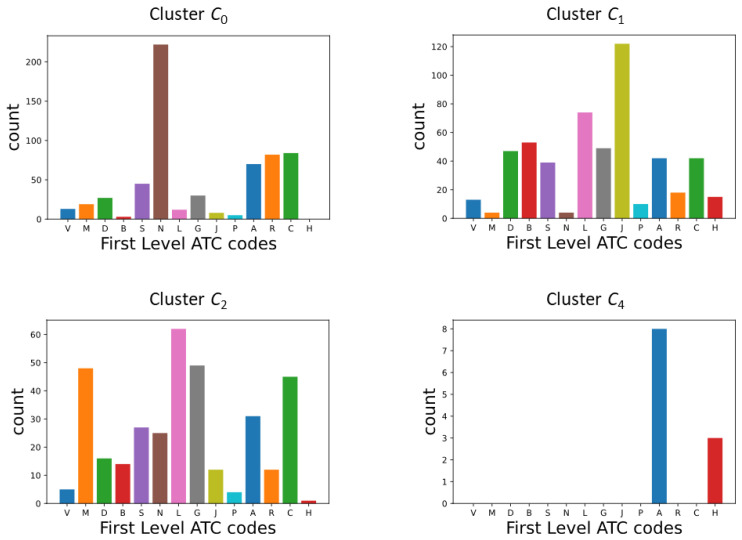
Histograms of level 1 ATC codes in the DrugBank 5.1.8 DDSN clusters holding drug repositionings confirmed by literature review: cluster $C_{0}$ (brown nodes), cluster $C_{1}$ (green nodes), cluster $C_{2}$ (light blue nodes), and cluster $C_{4}$ (pink nodes). The dominant property in cluster $C_{0}$ is N–*Nervous System*, J–*Anti-infectives for systemic use* in cluster $C_{1}$, L–*Antineoplastic and immunomodulating agents* in cluster $C_{2}$, and A–*Alimentary Tract and Metabolism* in cluster $C_{4}$.

The histograms showing the dominant properties (as level 1 ATC codes) in clusters $C_{0}$, $C_{1}$, $C_{2}$, and $C_{4}$ are presented in Appendix B.2, Figure A9.

### 3.3. Repositioning Confirmations

#### 3.3.1. Confirmed Drug Repositionings in DrugBank 5.0.9

This section discusses the drug repositioning hits generated with our methodology in DrugBank 5.0.9 and confirmed with the level 1 ATC codes in DrugBank 5.1.8. Our procedure confirmed the predicted hints in modularity classes 0 and 2.

##### Modularity Cluster $C_{0}$

In modularity cluster $C_{0}$, DrugBank 5.1.8 confirms mepolizumab and naloxone (see Figure A1 and Figure A2). Naloxone (ATC code V03AB15) is a µ-opioid receptor antagonist indicated in the treatment of opioid overdose. In DrugBank 5.0.9, naloxone’s first level ATC is V–*Various*; its level 4 (V03AB) means naloxone is in the *Antidotes* category.

Our methodology predicts naloxone’s level 1 ATC as N–*Nervous system*; the latest DrugBank 5.1.8 adds two N level 1 ATC codes to naloxone (level 4 ATC category *Natural opium alkaloids* for the combinations with hydromorphone and oxycodone), thus confirming our prediction.

Mepolizumab (ATC code L04AC06) is a monoclonal antibody acting as an antagonist of interleukin-5, included in the L–*Antineoplastic and immunomodulating agents* level 1 ATC category by DrugBank 5.0.9.

DrugBank 5.1.8 does not list the L04AC06 code anymore for mepolizumab; instead, it uses the level 1 ATC code R–*Respiratory system* (the level 4 ATC is R03DX, which includes *other systemic drugs for obstructive airways diseases*, as mepolizumab is indicated in severe eosinophilic asthma).

##### Modularity Cluster $C_{2}$

In modularity cluster $C_{2}$, DrugBank 5.1.8 confirms torasemide, methazolamide, acetazolamide, dorzolamide, brinzolamide, zonisamide, and quinetazone (see Figure A3).

Torasemide, quinetazone, methazolamide, acetazolamide, dorzolamide, and zonisamide, brinzolamide (ATC codes: C03CA04, C03BA02/C03BB02, S01EC05, S01EC01, S01EC03, N03AX15, S01EC04/S01EC54) are sulfonamide compounds with various pharmacodynamic effects. According to DrugBank 5.0.9, torasemide and quinetazone are diuretics used as antihypertensive drugs, included in the C—*Cardiovascular system* level 1 ATC category. Zonisamide is an antiepileptic drug (level 1 ATC N–*Nervous system*). Methazolamide, acetazolamide, dorzolamide, and brinzolamide are carbonic anhydrase inhibitors used in glaucoma (level 1 ATC S–*Sensory organs*).

Our methodology predicts G–*Genito urinary system and sex hormones* as the level 1 ATC code for torasemide, quinetazone, methazolamide, acetazolamide, dorzolamide zonisamide, and brinzolamide. Indeed, the latest DrugBank 5.1.8 version includes all these drugs in the G level 1 ATC category—more precisely, in the G01AE level 4 ATC category of *Anti-infective and antiseptics* having a sulfonamide-based chemical structure.

#### 3.3.2. Drug Repositioning Hints in DrugBank 5.1.8

This section discusses the validity of some drug repositioning hints generated with our methodology in DrugBank 5.1.8; as this is the latest database version, we cannot use the same confirmation procedure based on ATC codes. Consequently, we provide evidence found in the state-of-the-art literature as confirmation clues. However, as both the number of clusters and their size prohibit an exhaustive literature search, we focus on the clusters with confirmed drug repurposing candidates—clusters $C_{0}$, $C_{1}$, $C_{2}$, $C_{4}$, and $C_{25}$.

Pyridoxal phosphate (cluster $C_{0}$, ATC code A11HA06) is the active form of vitamin B6 and belongs to the A–*Alimentary tract and metabolism* level 1 ATC category, along with the rest of water-soluble and fat-soluble vitamins. Our method predicts pyridoxal phosphate as level 1 ATC code N–*Nervous system* (see Figure 6); H-S Wang et al. reported that pyridoxal phosphate controls idiopathic intractable epilepsy in children [44]. P.B. Mills and team identified two groups of patients with neonatal epileptic encephalopathy (determined by PNPO mutations) that respond to pyridoxal phosphate [45].

Albendazole (cluster $C_{1}$, ATC code P02CA03) is an antiparasitic drug (first level ATC P–*Antiparasitic products, insecticides and repellents*) efficient in various helminthic infections. Our methodology predicts J as level 1 ATC code, suggesting potential systemic anti-infective effects (see Figure 7). Of note, ATC lists drug classes such as antivirals, antibacterials, antimycotics, and vaccines in the J–*Anti-infectives for systemic use* category. In vitro results show that albendazole exerts antifungal activity against *Aspergillus* spp. [46]; moreover, experiments on mice revealed antifungal effects against *Pneumocystis carinii* [47], confirming the new potential antifungal medical use of albendazole.

Methotrexate (cluster $C_{1}$, ATC codes L04AX03, L01BA01) is an anticancer and immunosuppressant agent; therefore, the level 1 ATC is L–*Antineoplastic and immunomodulating agents*. We predict the first level J–*Anti infectives for systemic use* (see Figure 7). The literature survey reveals several papers reporting in vitro antiviral effects of methotrexate in a dose-dependent manner on SARS-CoV-2 [48] and Zika virus replication [49]; methotrexate also prevents the replication of human cytomegalovirus and inhibits viral DNA synthesis [50].

Simvastatin, fluvastatin, lovastatin, and atorvastatin (cluster $C_{1}$, ATC codes A10BH51/C10AA01/C10BX04/C10BA02/C10BX01/C10BA04, C10AA04, C10AA02/C10BA01, and C10BX15/C10AA05/C10BX03/C10BA05/C10BX11/C10BX08/C10BX06/C10BX12) are HMG-CoA reductase inhibitors (also called statins) that lower serum lipid levels, reducing the risk of cardiovascular events caused by hyperlipidemia; they are in the level 1 ATC C–*Cardiovascular system* class. The first level of their ATC code, as predicted by our method, is J–*Anti infectives for systemic use* (see Figure A5), confirmed by literature; as such, simvastatin exhibits in vitro antimicrobial effect on methicillin-susceptible Staphylococcus aureus [51]. S.P. Parihar et al. [52] review the literature reporting preclinical and clinical evidence of statins effects in viral, parasitic, fungal, and bacterial infections, pointing out the factors that influence the response to statins, such as human polymorphism, metabolism, and drug interactions; this review includes data on all mentioned statins. Our algorithm predicts that all statins in cluster $C_{1}$ are potential anti-infective agents. As shown, for the statins we highlighted in Figure A5, we found literature confirming our prediction; for the other statins, new experiments and studies may provide confirmation.

Theophylline (cluster $C_{2}$, ATC codes R03DA54, R03DA74, R03DA20, R03DA04, and R03DB04) is a methylxanthine derivative used to treat obstructive respiratory conditions, such as asthma and COPD, hence having R–*Respiratory system* as first level ATC code. Our methodology indicates theophylline’s *Anticancer and immunomodulating properties*, as reflected by the predicted ATC first level L (see Figure A7), thus further confirming the repositioning proposed by our previous research [14]. Indeed, recent literature demonstrates the anticancer properties of theophylline in breast and cervical cell lines [53].

Meloxicam (cluster $C_{2}$, ATC codes M01AC56 and M01AC06) is an oxicam derivative with anti-inflammatory and antirheumatic properties of the M–*Musculo-skeletal system* ATC category. Our network-based methodology predicts L as the first level of the ATC code (see Figure A6). The literature confirms our prediction of the anticancer properties of meloxicam: Meloxicam inhibits tumor growth in COX-2 positive colorectal cancer [54]. Tsubouchi et al. report that COX-2 plays a significant role in the pathogenesis and progression of non-small cell lung cancer (NSCLC), demonstrating the inhibitory effect of meloxicam on the NSCLC growth by preferentially inhibiting COX-2 [55]. Reference [56] shows that meloxicam is efficient in osteosarcoma in both COX-2-dependent and independent inhibitory manners.

Cholecalciferol, ergocalciferol, and calcifediol (cluster $C_{2}$, ATC codes M05BB09/M05BX53/M05BB07/M05BB08/A11CC55/M05BB05/A11CC05/M05BB03/M05BB04, A11CC01, and A11CC06) are vitamin D analogs. Cholecalciferol (vitamin D3) is a fat-soluble vitamin (ATC level 1 A–*Alimentary tract and metabolism*, a category which includes hydro-soluble and lipo-soluble vitamins) with a well-established role in bone mineralization (ATC second level M05–*Musculo-skeletal system, drugs for treatment of bone diseases*). Ergocalciferol and calcifediol are also grouped in A–*Alimentary tract and metabolism* level 1 ATC. We predict these drugs as targeting diseases at level 1 ATC code L–*Antineoplastic and immunomodulating agents* (see Figure 8).There is extensive literature reporting the beneficial effects of vitamin D analogs in different cancers and highlighting the epidemiological, preclinical, and clinical results; all these back up their evolution as prophylactic and curative anticancer drugs [57,58].

Chloroquine (cluster $C_{2}$, ATC code P01BA01) is an antimalarial drug; consequently, it belongs to the P–*Antiparasitic products, insecticides and repellents* level 1 ATC category. According to our results, the predicted first-level ATC is L–*Antineoplastic and immunomodulating agents* for chloroquine (dominant in cluster $C_{1}$, see Figure A8). Multiple research reviews report in vitro, in vivo, and clinical trials testing chloroquine’s anticancer effect in glioblastoma [59] and other types of cancers [60,61,62,63], hence supporting the potential repositioning of chloroquine as an anticancer drug, as uncovered by our methodology.

Mecasermin and mecasermin rinfabate (cluster $C_{4}$, ATC codes H01AC03, H01AC05) are recombinant insulin-like growth factor-1 drugs indicated in growth failure in children with primary IGF-1 deficiency and, hence, are included in the H–*Systemic hormonal preparations, excluding sex hormones and insulins*. Literature and medicine regulatory authorities reports present the secondary pharmacologic actions of mecasermin and mecasermin rinfabate, including the anabolic and insulin-like effects (i.e., hypoglycemia) [64,65,66]; these pharmacologic effects could place the drugs in the A–*Alimentary tract and metabolism* level 1 ATC, as predicted by our methodology (see Figure 9).

Ornithine (cluster $C_{25}$, ATC code A05BA06) is a non-essential amino acid indicated as nutritional supplementation and for a good liver function and included in the A–*Alimentary tract and metabolism* level 1 ATC. M. Miyake et al. suggest that L-ornithine may interfere with the Central Nervous System, following a randomized, double-blind controlled trial that demonstrated that L-ornithine relieved stress and improved sleep quality in humans compared to the placebo group [67]. Indeed, we predicted ornithine at level 1 ATC N–*Nervous system* (see Figure 10).

## 4. Discussion

In this section, we discuss the particularities of our method, namely the data we use, the limitations of our method and its validation with ATC codes, and the way to integrate it into an ensemble drug repositioning framework.

### 4.1. Drug–Gene Interactions

The method we propose in this paper uses drug–gene interaction data from DrugBank versions 5.0.9 and 5.1.8. Table 2 presents examples of drug–gene interactions and their corresponding types, as defined by DrugBank 5.1.8 (see a detailed list of drug–gene interaction types in the Supplementary Materials Table S1 file *DDSN-results.xls* and how to retrieve such drug–gene interactions from DrugBank in the GitHub page https://github.com/GrozaVlad/Drug-repurposing-using-DDSNs-and-modularity-clustering (last commit on 21 October 2021)).

### 4.2. Method Limitations

The mechanisms that influence the polypharmacological profile of drugs are highly complex. Indeed, the medicinal compound interacts with a complex system represented by the human organism. Complex systems are context-dependent; in other words, any detail at the micro-scale influences the macroscale behavior. As such, many factors can be considered when analyzing the functions of any pharmaceutical substance: from the chemical structure to various types of relationships and interactions, as well as pharmacokinetics and pharmacodynamics. By this logic, our approach is limited to considering a narrow informational angle, namely drug–gene interactions. Nonetheless, considering many mechanisms and types of data simultaneously within the same model would be prohibitively complex, and the networks would become much too dense for any centrality of community analysis. Even considering one type of information has become significantly complex; for instance, the drug–drug interaction networks in DrugBank 3.0 had an average degree of ∼20, and in DrugBank 5.1.8 the average DDI network degree is ∼600). Recent literature [68,69,70] advances the so-called ensemble methods to address this new situation of being confronted with an overabundance rather than scarcity of data (see Section 4.4).

### 4.3. Labeling and Validation with ATC Codes

Employing computational methods (i.e., data mining and machine learning) in drug repositioning is generally hampered because we do not a have robust ground truth. Indeed, databases such as DrugBank record positive information about the drugs’ known properties and functions, yet the absence of evidence is not evidence of absence (some drug properties may be hidden, and only future experiments can fully reveal them). That is why performance evaluation and validation of computational drug repositioning models are still an open issue; therefore, researchers adopt ad hoc, particular strategies, which are hard to compare [71]. Consequently, we resorted to making predictions with an older database version and then validating them with the latest version. However, even the latest database still cannot contain exhaustive information about drug functions. Furthermore, the negative information on drug functions/effects (stating what properties a drug does not have) will help prune the vast search space in drug repositioning. Unfortunately, negative information is scarce and scattered throughout the literature; to the best of our knowledge, no comprehensive dataset contains such data based on experimental results. As such, the existing negative information cannot be used algorithmically/automatically. As explained, one feasible method for filtering the noise and navigating the search space affected by uncertainty—an approach supported by recent research—is to integrate tools (such as the one we propose here) in ensemble methods.

Many computational drug repositioning methods based on complex networks rely on community detection and community labeling. However, labeling can be cumbersome and subjective; thus, we decided to use ATC codes, since this system is the standard for classifying medicines accepted by the WHO. Furthermore, the automated approach is fostered because the ATC code aggregates all information about a drug in a combination of letters and numbers, which are easier to process algorithmically. The ATC code classifies drugs on five levels considering three criteria simultaneously: anatomical (A)—the first level; therapeutic (T)–levels 2 and 3; and chemical (C)—levels 4 and 5. The anatomical criterion indicates the anatomical level or the physiological organ systems on which a specific drug acts. Each anatomical level is indicated in the ATC code by a letter (e.g., A–*Alimentary tract and metabolism*, C–*Cardiovascular system*, M–*Musculoskeletal system*, or R–*Respiratory system*); the ATC system contains 14 anatomical groups. Level 2 represents the therapeutic classification criterion and is encoded by two digits. Level 3 (encoded by a letter) indicates the particular pharmacological group of the drug. Level 4 (encoded by a letter) indicates the chemical class of the drug. Level 5 is encoded by two digits the chemical structure of the drug. This paper only used the first-level ATC codes for labeling and validation of prediction, although drug function is more precisely expressed by levels 1–3; we opted perform this because the sophisticated hierarchical clustering algorithms entailed by such an approach would have unnecessarily intensified the computational character of our study.

### 4.4. Method Application

When the problem at hand is too complex to solve by employing a single model, machine learning uses an ensemble strategy [72], which trains several models on the same set of data to operate collectively for solving the problem. This strategy is already used in bioinformatics to approach complex problems such as motif discovery in ChIP-Seq data [73]. The problem of drug repositioning is also very complex; however, prediction accuracy is not the primary indicator of success (the benefit of correctly predicting even a few drug repositionings is more significant than the cost of experiments entailed by testing the wrong predictions [74].) As such, very recent literature advances the idea of using ensemble methods for drug repositioning [69,70].

**Figure 11:**
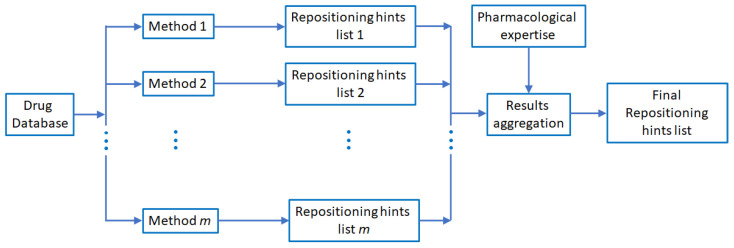
Overview of the ensemble strategy in drug repositioning. A group of machine learning and data mining methods $\left\{\mathrm{Method}1,\mathrm{Method}2,\ldots \mathrm{Method}m\right\}$, implementing various models and using distinct features (e.g., drug–drug interactions, drug–target interactions, drug–gene interactions, drug–adverse reactions relationships, pharmacokinetic properties) from the same comprehensive dataset and predicting a list of drug repositioning hints. Each method Methodi generates its repositioning hints list, and an aggregation process assembles all lists in the final repurposing hints list.

In this context, considering that—as explained in Section 4.2—our method uses drug–gene interaction data that partially describes the behavior of drugs, we indicate the ensemble strategy as ta method to use our method. As shown in Figure 11, drug repositioning prediction based on drug–gene interaction data may be Methodi from the group of machine learning methods based on distinct models $\left\{\mathrm{Method}1,\mathrm{Method}2,\ldots \mathrm{Method}m\right\}$. The repositioning hints list *i* is aggregated (i.e., via voting, averaging, or other procedures) to produce a final drug repositioning hints list. The aggregation process may use pharmacological expertise, e.g., to adjust the weights of a weighted average. However, implementing the ensemble strategy is beyond the scope of this paper, which aims to analyze and promote—for the first time—the beneficial role of drug–gene interaction networks for computational drug repositioning.

## 5. Conclusions

In this paper, we propose a new drug repurposing methodology based on algorithmic complex network analysis. To this end, we introduce an original method of building the Drug–Drug Similarity Network (DDSN) using drug–gene interactions from DrugBank, clustering DDSN with modularity classes, and labeling each cluster with the dominant first level ATC code of drugs within the cluster. The assumption that results in drug repurposing hints is that drugs in a cluster share the dominant property of the cluster. We use an automated procedure to tune modularity resolution, to apply our methodology on a DDSN built with data from DrugBank 5.0.9, to generate the list of drug repurposing hints (i.e., drugs for which the first level ATC does not match the dominant cluster label), and to check it against ATC codes in DrugBank 5.1.8.

By running our method on the DrugBank 5.1.8 DDSN, we generated a consistent list of drug repositioning candidates; we select the top betweenness/degree drugs in each cluster and perform a preliminary validation with state-of-the-art experimental results reported in the literature. Due to the fact that we collected many literature confirmations of our method’s predictions, we argue that our fully automated pipeline, based on Big Data and unsupervised machine learning, is a practical tool that can substantially narrow the enormous search space in drug repositioning.

To summarize, the overarching methodological contributions of our paper are listed as follows:

(i) A new method to build weighted drug–drug similarity networks based on drug–gene interactions;
(ii) An automated procedure to optimize the modularity resolution such that network clustering maximizes the number of identified drug repurposings. A known/ confirmed drug repurposing is a drug with more level 1 ATC codes in the latest drug database, compared with the earlier database—used to generate the drug–drug similarity network;
(iii) A new drug repurposing list was generated with our pipeline from the latest DrugBank 5.1.8 by analyzing the three most representative clusters.

In the present context, affected by the COVID-19 pandemic, we believe that the most promising findings/results presented in our paper are the anti-infective effects of statins, especially their potential antiviral effects. Indeed, the very recent comprehensive study [6] also finds, following in vitro screening, that fluvastatin presents what the authors call “strong effect” against SARS-CoV-2.

Considering all aspects presented in Section 4.2, we will extend our research on drug–gene interaction networks by implementing hierarchical clustering to predict ATC codes on levels 1–3, developing a dedicated cluster overlapping algorithm as a drug repositioning prediction strategy (i.e., one would reasonably expect that drugs in the overlapping zone would inherit the dominant properties of the respective clusters) and integrating the drug–gene network method into an ensemble strategy. These future objectives require substantial reliance on developing bioinformatic tools, entailing algorithm design, machine learning, and Big Data analytics.

## Figures and Tables

**Table 1:** The list of drug repurposing candidates generated with our methodology in Figure 1 on data from DrugBank 5.1.8, and confirmed with scientific literature. The rows correspond to drugs or drug classes (for example, simvastatin, fluvastatin, lovastatin, and atorvastatin are statins). The columns indicate—from left to right—the name, the cluster, the current level 1 ATC code in DrugBank 5.1.8, the predicted level 1 ATC code, and the confirmation references for the drug (or drug class) in each row.

Drug	Cluster	Current Level 1 ATC	Predicted Level 1 ATC	References
Pyridoxal phosphate	$C_{0}$	A	H	[44,45]
Albendazole	$C_{1}$	P	J	[46,47]
Methotrexate	$C_{1}$	L	J	[48,49,50]
$\left[\begin{array}{l} \mathrm{Simvastatin}\\ \mathrm{Fluvastatin}\\ \mathrm{Lovastatin}\\ \mathrm{Atorvastatin} \end{array}\right.$	$C_{1}$	C	J	[51,52]
Theophylline	$C_{2}$	R	L	[14,53]
Meloxicam	$C_{2}$	M	L	[54,55,56]
$\left[\begin{array}{l} \mathrm{Cholecalciferol}\\ \mathrm{Ergocalciferol}\\ \mathrm{Calcifediol} \end{array}\right.$	$C_{2}$	M, A	L	[57,58]
Chloroquine	$C_{2}$	P	L	[59,60,61,62,63]
$\left[\begin{array}{l} \mathrm{Mecasermin}\\ \mathrm{Mecasermin rinfabate} \end{array}\right.$	$C_{4}$	H	A	[64,65,66]
Ornithine	$C_{25}$	A	N	[67]

**Table 2:** Examples of drug–gene interactions listed in DrugBank.

Drug Name	Gene Name	Interaction Type
Alteplase	PLG	activator
Hydromorphone	OPRK1	agonist
Varenicline	CHRNB2	partial agonist
Prazosin	ADRA1B	antagonist
Ascorbic acid	EGLN1	chaperone
Pyridoxal phosphate	GAD1	cofactor
Vardenafil	PDE6G	allosteric modulator
Trastuzumab	ERBB2	antibody
Nusinersen	SMN2	antisense oligonucleotide
Methysergide	HTR1F	binder
Tiapride	DRD2	blocker
Carvedilol	KCNJ4	inhibitor
Clobetasol propionate	ANXA1	inducer
Clofazimine	PPARG	modulator
Cerliponase alfa	IGF2R	ligand
Filgrastim	CSF3R	stimulator
Dalteparin	SERPINC1	potentiator
Vitamin A	RDH13	substrate
Nedocromil	CYSLTR1	suppressor
Belimumab	TNFSF13B	neutralizer
Esmirtazapine	HRH1	inverse agonist
Procainamide	DNMT1	other
Haloperidol	HTR2A	other/unknown

## Data Availability

This study uses only public database data.

## Supplementary Materials

The following are available online at https://www.mdpi.com/article/10.3390/pharmaceutics13122117/s1, Table S1: DDSN-results.

## Abbreviations

The following abbreviations are used in this manuscript:

ATC	Anatomical Therapeutic Chemical;
COPD	Chronic Obstructive Pulmonary Disease;
COX-2	Cyclooxygenase-2;
DDSN	Drug–Drug Similarity Network;
NSCLC	Non-Small Cell Lung Cancer.
